# Validity of the Rapid Thyroxine Absorption Test for the Differentiation Between Levothyroxine Non-compliance and Malabsorption in Thyroid-Stimulating Hormone Refractory Hypothyroidism

**DOI:** 10.7759/cureus.37776

**Published:** 2023-04-18

**Authors:** Akram A Alhassan, Haider A Alidrisi, Abbas A Mansour

**Affiliations:** 1 Diabetes and Endocrinology, University of Basrah, College of Medicine, Basrah, IRQ; 2 Diabetes and Endocrinology, Faiha Specialized Diabetes, Endocrine, and Metabolism Center, Basrah, IRQ

**Keywords:** levothyroxine non-compliance, levothyroxine malabsorption, thyroid-stimulating hormone refractory hypothyroidism, long levothyroxine absorption test, rapid thyroxine absorption test, hypothyroidism

## Abstract

Introduction

Thyroid-stimulating hormone refractory hypothyroidism is a common problem. This is due to either non-compliance or malabsorption with levothyroxine (LT4). The study aimed to assess the validity of the rapid LT4 absorption test in the differentiation between LT4 malabsorption and non-compliance.

Methods

A cross-sectional study was done from January to October 2022 at Faiha Specialized Diabetes, Endocrine, and Metabolism Center, in Basrah, Southern Iraq. Twenty-two patients with thyroid-stimulating hormone (TSH) refractory hypothyroidism were evaluated by rapid LT4 absorption test with measurements of TSH before 1000 μg LT4 intake, and free thyroxine (pmol/l) and total thyroxine before (nmol/l) (baseline TT4 and baseline FT4) and two hours after (2-HR TT4 and 2-HR FT4). The findings were compared with the following four-week-long supervised LT4 absorption test results.

Results

In the rapid LT4 absorption test, patients with (2-HR FT4 minus baseline FT4 ≤1.28 pmol/l (0.1 ng/dl) or 2-HR FT4 minus baseline FT4 1.28-6.43 pmol/l (0.1-0.5 ng/dl) plus 2-HR TT4 minus baseline TT4<72.08 nmol/l (5.6 µg/dl)), eight out of 10 patients were correctly diagnosed with malabsorption. And in those with (2-HR FT4 minus baseline FT4 ≥6.43 (0.5 ng/dl) or 2-HR FT4 minus baseline FT4 1.28-6.43 (0.1-0.5 ng/dl) plus 2-HR TT4 minus baseline TT4≥72.08 (5.6 µg/dl)), 11 out of 12 patients were correctly diagnosed as non-compliant. This criterion showed 88.8% sensitivity, 15.4% specificity, 80% positive predictive value, and 91.6% negative predictive value for diagnosing LT4 malabsorption.

Conclusion

The rapid LT4 absorption test showed good diagnostic accuracy in differentiating non-compliance from malabsorption when (2-HR FT4 minus baseline FT4) and (2-HR TT4 minus baseline TT4) were used as criteria.

## Introduction

In adults, hypothyroidism is the most common thyroid disorder, and it is more common in females and with increasing age. It has a wide diverse presentation ranging from asymptomatic to severe forms presenting as myxedema coma, a life-threatening complication [1,2]. The synthetic thyroid hormone levothyroxine (LT4) is an affordable treatment for hypothyroidism with few adverse effects [3]. Persistence of hypothyroid symptoms in patients on LT4 in a dose more than 1.9 µg/kg body weight or thyroid stimulating hormone (TSH) more than 4.5 µU/ml after six weeks of dose increase is called TSH refractory hypothyroidism [4]. Non-compliance is attributed to multiple factors and limitations imposed by therapy; the patient should take the medication in a fasting state, the period between medication and meal, the daily basis of medication, and avoidance of multiple drugs familiar to the patient which interfere with LT4 absorption [5]. Weekly administration of LT4 is an alternative strategy to a daily dose of LT4 intended to improve the adherence of patients. In addition, there is resistance to conventional doses of oral LT4 in patients with inflammatory bowel disease, celiac disease, lactose intolerance, atrophic gastritis [6-9], and Helicobacter pylori infection [10]. Interference with LT4 absorption is caused by taking some foods and drinks including fibers, soy protein, coffee, and grapefruit [11,12]. Moreover, some drugs interfere with LT4 absorption when co-administered with them. Examples of these drugs are calcium elements, ferrous components, aluminum hydroxide, chromium picolinate, cholestyramine, colesevelam, gastric protectants like sucralfate, estrogen receptor inhibitors like raloxifene, and phosphate chelators like sevelamer [13].

Duodenum and jejunum are the absorption sites of 60-80% of administered LT4 doses, with maximum absorption occurring when the stomach is empty and within three hours of ingestion [14]. The gastric power of hydrogen (pH) is essential for LT4 absorption. Therefore, proton pump inhibitors, gastrectomy, and gastric bypass surgery result in LT4 malabsorption [15].

Patients who continue to suffer from biochemical and/or clinical hypothyroidism despite a high dose of LT4 may be described as non-compliance or pseudo malabsorption, a condition that is usually due to non-adherence to medications. Some of these patients may have underlying psychiatric disorders, but not a simple matter of therapy abandonment [16].

Even after careful consideration of all factors that lead to malabsorption, still there are difficulties to differentiate malabsorption from non-compliance. A large single dose and a weekly dose of LT4 are two protocols used to discriminate malabsorption from non-compliance in TSH refractory hypothyroidism [17,18].

The study aimed to assess the validity of the rapid LT4 absorption test for the differentiation between non-compliance and malabsorption in TSH refractory hypothyroidism.

## Materials and methods

A cross-sectional study was conducted at Faiha Specialized Diabetes Endocrine and Metabolism Center (FDEMC), Basrah, Southern Iraq, during the period from January 2022 to October 2022. Through the period of the study, 537 out of 1120 patients with hypothyroidism had visited the center for follow-up and had TSH above target. Clinical data were taken in the form of age, duration of hypothyroidism, cardiovascular disease, malabsorption diseases, history of thyroidectomy, and drug history. Body weight and height were measured in bare feet and light clothes for calculation of body mass index (BMI) in kilogram/squared meter (kg/m2).

Inclusion and exclusion criteria

We included patients aged 18-65 years with TSH refractory hypothyroidism who were currently taking LT4 in a dose of >1.9 µg/kg and TSH >4.5µIU/ml after six weeks of dose increase. The patients agreed to participate in the study to be involved in both the rapid and supervised long LT4 absorption protocols. 

The patients were excluded if they had a history of the following conditions: cardiovascular disease, pregnancy, current use of drugs that interfere with levothyroxine absorption or oral contraceptive pills, history of malabsorption syndrome, and declared non-compliance with LT4 intake.

After evaluation, 34 patients met the inclusion criteria and agreed to participate in the study. A written informed consent was taken from each participant in accordance with the ethical standards of the FDEMC Research Committee, from which ethical approval was obtained (ref #62/38/21), and with the 1964 Declaration of Helsinki and its later amendments or comparable ethical standards.

Hormonal assay

The fully automated chemiluminescence immunoassay cobas e411 platform (Roche, Basel, Switzerland) was used for the measurement of serum TSH (normal range 0.27-4.2 µIU/ml), serum free thyroxine (FT4) (normal range 11.9-21.8 pmol/l, 0.93-1.7 ng/dl), and serum total thyroxine (TT4) (normal range 65.6- 181.5 nmol/l, 5.1- 14.1µg/dL).

Rapid LT4 absorption test

After an overnight fast, early morning, and with an empty stomach, each patient was given 1000 µg LT4 orally and supervised over the next hour for vomiting and any clinical deterioration. Two blood samples were taken from each patient (five ml for each sample), put in a clot activator tube, centrifuged immediately, and serum separated for analysis. The first sample (baseline) was taken before LT4 intake for analysis of TSH, FT4, and TT4. The second sample was taken two hours later (2-HR) for analysis of FT4 and TT4. The evaluations of these samples were done by the following criteria:

Percentage of LT4 absorption was calculated by the following equation [19]: Volume of Distribution (VD) = 0.442 x BMI. Percent absorbed = (Peak TT4- Baseline TT4) µg/dL x 10 dl/L x VD L / Total administered LT4 dose (µg). Normal % absorbed range 50-100%, Average 80%. Peak absorption of less than 60% indicates LT4 malabsorption.

2-HR FT4/baseline FT4 ratio: a ratio of ≥ 2.5 was considered as normal response (non-compliance) [20]. Further assessments using the increment in FT4 which is 2-HR FT4 minus baseline FT4 and the increment in TT4 which is 2-HR TT4 minus baseline TT4.

Supervised long LT4 absorption test

This test was considered the standard test for differentiating non-compliance from malabsorption in the study. It was started in each patient on the seventh day after the rapid LT4 absorption test. The total weekly dose of LT4 (1.9 µg/kg body weight multiplied by seven) was given each week for the next four weeks in the early morning on an empty stomach and the patients were supervised for the next two hours for vomiting or any clinical deterioration. At the end of the period of four weeks later, TSH measurements were done again. Based on the results, the patients were classified into two groups, non-compliance (TSH ≤4.2 µIU/ml) and malabsorption (TSH >4.2 µIU/ml).

Both tests were well tolerated by all participants without any serious side effects. However, only 22 patients completed the supervised long LT4 absorption test and were involved in the final analysis. Missing at least one-week appointment was considered an exclusion from the final analysis.

Statistical analysis

The data were analyzed by the Statistical Package for the Social Sciences (SPSS), version 26.0 (IBM Corp., Armonk, NY, USA). Categorical variables were summarized as numbers (N) and percentages (%). Continuous variables were summarized as mean ± standard deviation (M ± SD). Comparisons of % absorption and 2-HR FT4/baseline FT4 ratio in between groups (malabsorption vs non-compliance) were done using the independent student t-test. Repeated-Measures Analysis of Variance (ANOVA) with a Greenhouse-Geisser correction was used to compare the values of FT4 and TT4 at baseline and after two hours of tests, and post hoc test with Bonferroni corrections for in between group comparisons. Receiver operating characteristic (ROC) curves were plotted for evaluation of the accuracy of (% absorption, 2-HR FT4/baseline FT4 ratio, 2-HR FT4 minus baseline FT4, and 2-HR TT4 minus baseline TT4) in the diagnosis of LT4 malabsorption. Correlations of different rapid LT4 absorption test cut-offs in between groups were done using Chi-square tests. A P-value of < 0.05 was defined as statistical significance for all above comparisons.

## Results

The general characteristics of the study patients are summarized in Table 1.

**Table 1:** General characteristics of the study of 22 patients. Abbreviations:  BMI, body mass index, LT4, levothyroxine, TSH, thyroid-stimulating hormone, TT4, total thyroxine, FT4, free thyroxine.

FT4 (pmol/l)	TT4 (nmol/l)	TSH (μIU/mL)	LT4 dose (μg)	BMI (kg/m^2^)	Duration (years)	Thyroidectomy	Age (years)	Gender	Patient
19.05	74.65	17.07	200	33	5	Yes	23	Male	1
14.03	101.56	29.35	200	41	6	No	22	Female	2
23.16	171.19	12	200	45	15	No	35	Female	3
12.09	144.93	18.91	250	30	10	Yes	34	Female	4
14.28	117.65	7.65	200	31	6	No	36	Female	5
14.03	101.56	29.35	200	41	11	No	22	Female	6
11.84	82.50	100.00	200	24	9	No	46	Female	7
15.70	149.31	24.93	250	26	2	No	19	Female	8
12.48	83.79	10.17	250	34	13	Yes	32	Female	9
14.41	93.32	23.93	250	38	11	No	41	Female	10
5.02	68.60	13.81	250	25	7	No	29	Female	11
5.92	44.40	100.00	300	33	4	Yes	30	Female	12
16.34	108.76	37.35	250	34	5	Yes	29	Male	13
7.72	51.48	24.27	200	29	9	Yes	44	Female	14
15.44	113.91	12.29	250	43	4	No	30	Female	15
0.12	67.76	12.62	300	54	6	No	46	Male	16
13.12	154.97	20.31	250	35	8	No	45	Female	17
14.93	100.01	16.04	250	31	2	Yes	35	Female	18
9.65	58.69	36.41	250	49	13	No	48	Female	19
13.00	105.03	44.25	200	32	2	No	18	Female	20
7.20	61.78	44.63	200	26	3	No	40	Female	21
8.36	85.59	150.00	250	34	5	Yes	39	Female	22
12.87±4.37	96.54±33.46	35.2±36	234.0±32.3	34.9±7.7	7 ± 3.8	9 (40.9%)	33.6 ±9.4	Female 19(96.4%)	M± SD Or N(%)

After a one-month follow-up with a long supervised LT4 absorption test, 13 (59%) were diagnosed as non-compliance and nine (41%) as having malabsorption.

**Figure 1:**
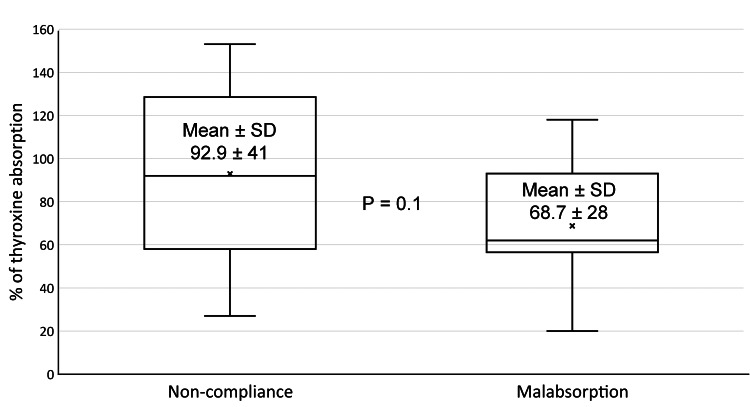
In between groups, comparisons of mean % absorption. Abbreviations: SD, standard deviation.

On analysis of the results of the rapid LT4 absorption test, the means of % absorption did not differ significantly in between groups, as shown in Figure 1.

**Figure 2:**
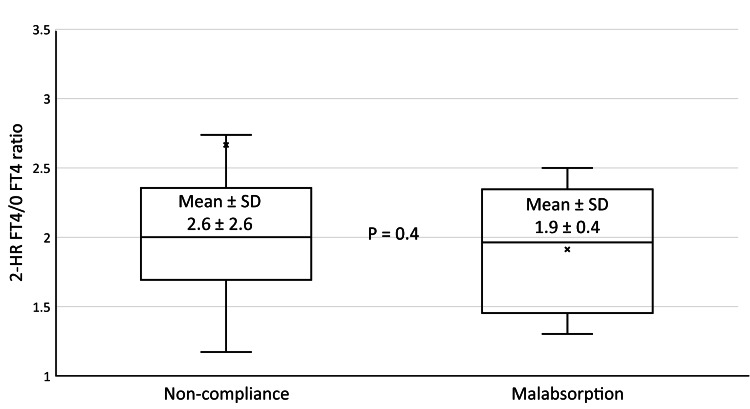
In between groups comparisons of mean 2-HR FT4/baseline FT4 ratio. Abbreviations: 2-HR FT4, two-hour free thyroxine, 0 FT4, baseline free thyroxine, SD, standard deviation.

A similar finding was also seen on comparison of the 2-HR FT4/baseline FT4 ratio that did not differ significantly in between groups, as shown in Figure 2.

**Figure 3:**
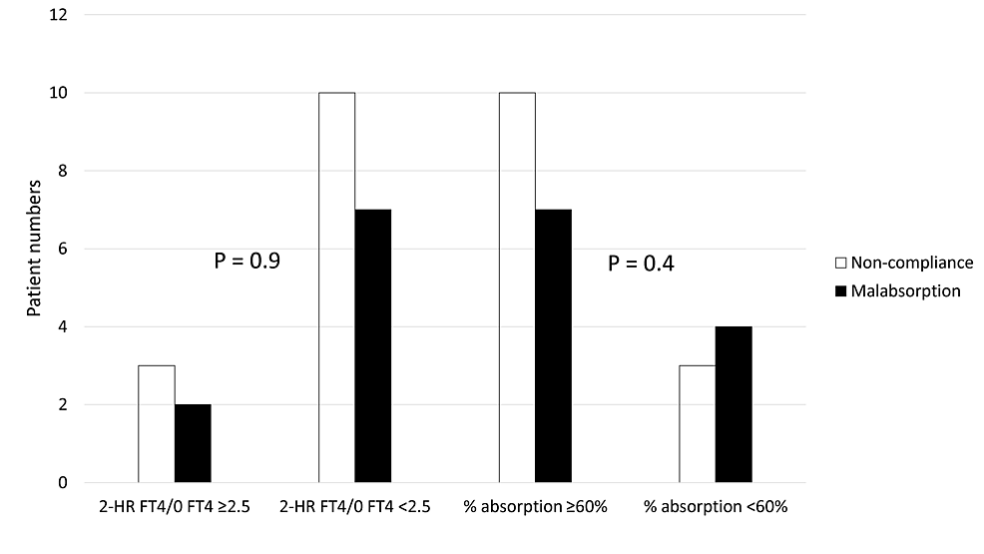
Chi-square test for both 2-HR FT4/baseline FT4 ratio ≥2.5 versus <2.5 and % absorption ≥60% versus <60% between the study groups. Abbreviations: 2-HR FT4, two-hour free thyroxine, 0 FT4, baseline free thyroxine.

It was also shown that both % absorption ≥60% and 2-HR FT4/baseline FT4 ratio ≥2.5 cut-off failed to differentiate between LT4 malabsorption and non-compliance as shown in Figure 3.

**Figure 4:**
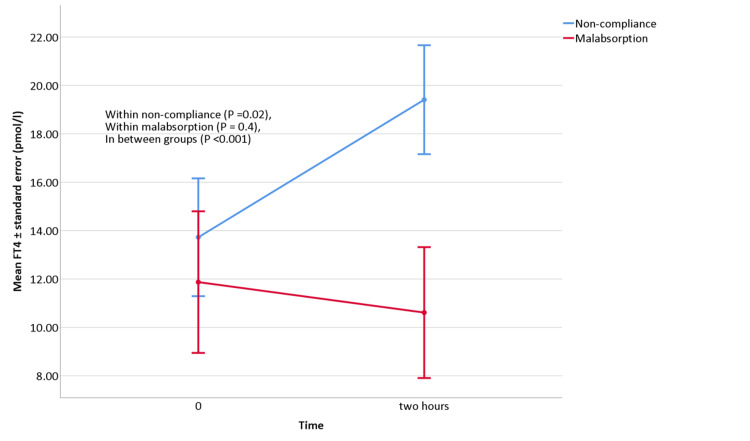
Repeated Measures ANOVA for rapid LT4 absorption test for FT4. Abbreviations: FT4, free thyroxine, LT4, levothyroxine

A Repeated Measures ANOVA showed that FT4 was significantly increased after two hours of the rapid LT4 absorption test in the non-compliance group (FT4 increment was 5.14±7.72 pmol/l, 95%CI (0.90-10.29), P = 0.02). The Malabsorption group showed no significant change (-1.15±5.14 pmol/l, 95%CI (-5.14-2.57), P = 0.4) (Figure 4).

**Figure 5:**
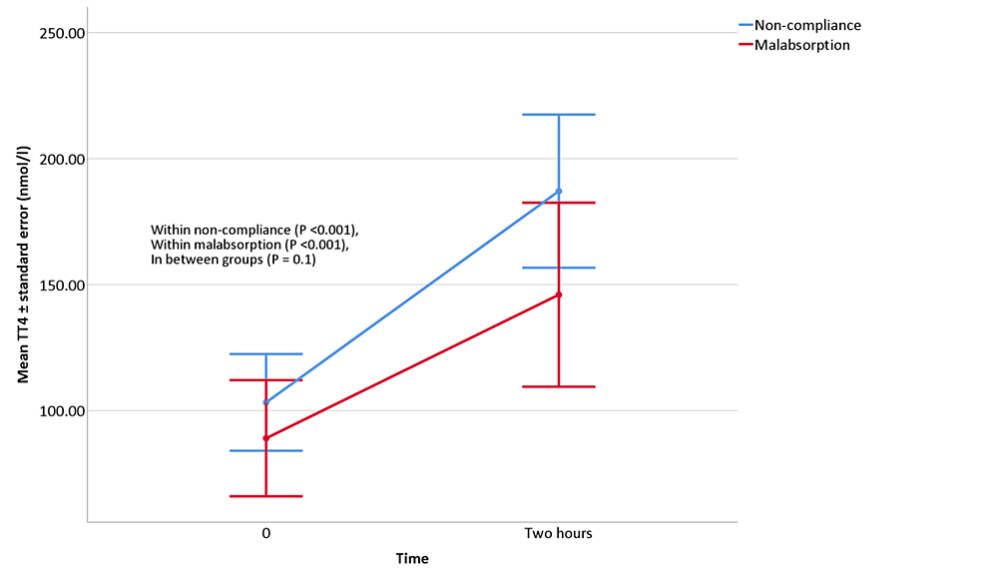
Repeated Measures ANOVA for rapid LT4 absorption test for TT4. Abbreviations: TT4, total thyroxine, LT4, levothyroxine

While for TT4, it increased significantly in both groups. Noncompliance patients (83.66±39.90 nmol/l, 95%CI (59.21-108.12), P < 0.001), malabsorption patients (56.63±28.31 nmol/l, 95%CI (34.75-78.51), as shown in Figure 5.

**Figure 6:**
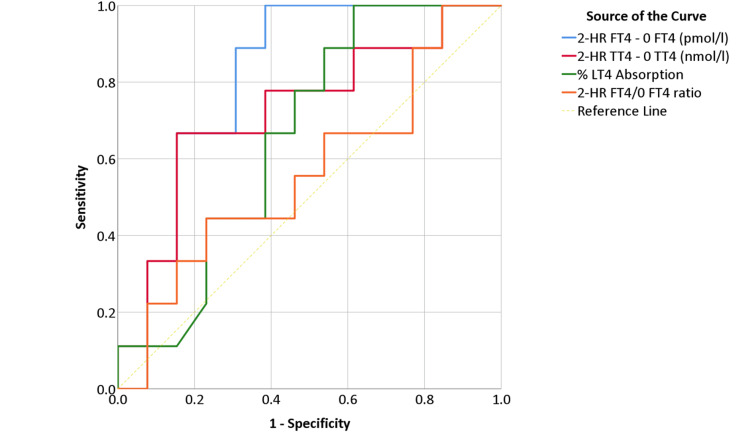
ROC curves of the different studied criteria for the diagnosis of LT4 malabsorption using the rapid LT4 thyroxine absorption test. Abbreviations: 2-HR FT4 – 0 FT4, two-hour free thyroxine minus baseline free thyroxine, 2-HR TT4 – 0 TT4, two-hour total thyroxine minus baseline total thyroxine, % LT4 absorption, percentage levothyroxine absorption, 2-HR FT4/0 FT4 ratio, two-hour free thyroxine / baseline free thyroxine ratio.

In this study, 2-HR FT4 minus baseline FT4 demonstrated a ROC curve with a good accuracy for the diagnosis of malabsorption (area under the curve (AUC) = 0.812, standard error = 0.095, P = 0.01, 95%CI (0.6-0.9)) as shown in Figure 6. The 2-HR FT4 minus baseline FT4 cut-off 6.43 pmol/l (0.5 ng/dl) and below had 100% sensitivity and 62% specificity, and the cut-off 1.28 pmol/l (0.1 ng/dl) and below had 66% sensitivity and 85% specificity for malabsorption. The 2-HR TT4 minus baseline TT4, % of absorption, and 2-HR FT4/baseline FT4 ratio did not demonstrate a significant ROC (P >0.05). The 2-HR TT4 minus baseline TT4 showed (AUC = 0.726, standard error = 0.115, P = 0.07, 95%CI (0.5-0.9). A cut-off of 72.08 nmol/l (5.6 μg/dl) and below had 77.8% and 62% sensitivity and specificity, respectively.

**Figure 7:**
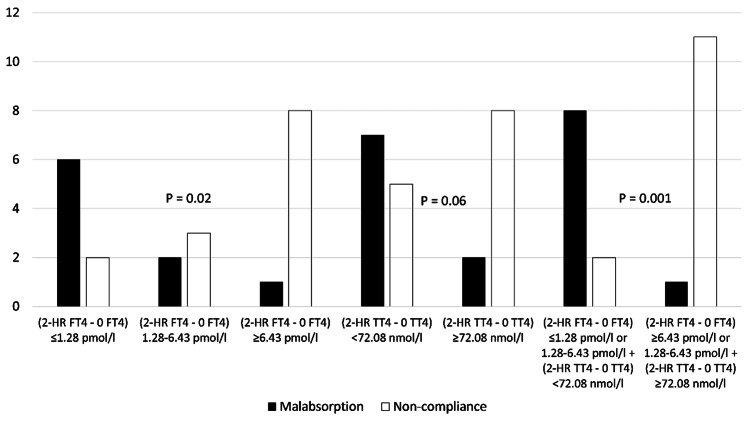
Comparison of the different cut-offs of two hours FT4 and TT4 increments in the rapid LT4 absorption test for the diagnosis of LT4 malabsorption. Abbreviations: 2-HR FT4 – 0 FT4, two-hour free thyroxine minus baseline free thyroxine; 2-HR TT4 – 0 TT4, two-hour total thyroxine minus baseline total thyroxine, LT4, levothyroxine

In the rapid LT4 absorption test, within 2-HR FT4 minus baseline FT4 ≤1.28 pmol/l (0.1 ng/dl), six out of eight were correctly diagnosed with malabsorption, and within 2-HR FT4 minus baseline FT4 ≥6.43 pmol/l (0.5 ng/dl), eight out of nine correctly diagnosed as non-compliant, P = 0.02. Using a cut-off 2-HR TT4 minus baseline TT4≥72.08 nmol/l (5.6 μg/dl), eight out of 10 were correctly diagnosed as non-compliant. When these two criteria merged, rapid LT4 absorption test results with (2-HR FT4 minus baseline FT4 ≤1.28 nmol/l (0.1 ng/dl) or 2-HR FT4 minus baseline FT4 1.28-6.43 pmol/l (0.1-0.5 ng/dl) plus 2-HR TT4 minus baseline TT4<72.08 nmol/l (5.6 μg/dl)), eight out of 10 were correctly diagnosed with malabsorption. On the other hand, (2-HR FT4 minus baseline FT4 ≥6.43 pmol/l (0.5 ng/dl) or 2-HR FT4 minus baseline FT4 1.28-6.43 pmol/l (0.1-0.5 ng/dl) plus 2-HR TT4 minus baseline TT4≥72.08 (5.6 μg/dl)), 11 out of 12 correctly diagnosed as non-compliant. These criteria showed 88.8% sensitivity, 15.4% specificity, 80% positive predictive value, and 91.6% negative predictive value for the diagnosis of LT4 malabsorption, as shown in Figure 7.

## Discussion

Poor adherence to daily administration of LT4 is the most common cause for non-responding to high doses of LT4 [21]. The LT4 absorption tests (different protocols) are used to differentiate between non-compliance and true malabsorption [17,18]. In the current study, we consider the long-supervised LT4 absorption test as a gold standard test for comparison with and validity assessment of other tests for multiple reasons. First, we gave a weekly dose of 1.9µg/kg LT4 under the supervision of a doctor to ensure compliance and safety. Second, TSH needs at least four to six weeks for normalization [22]. In the rapid LT4 absorption test, Soares et al. suggested that the ratio 2-HR FT4/baseline FT4 of more than or equal to 2.5 means LT4 non-compliance in TSH refractory hypothyroidism [20]. In the present study, neither the mean 2-HR FT4/baseline FT4 ratio nor its cut-off 2.5 could significantly differentiate between malabsorption and non-compliance. When LT4 % absorption was used as a criterion, a similar finding was seen with poor diagnostic accuracy for LT4 malabsorption. While a review of data from 16 patients from the Mayo Clinic showed that the peak % LT4 absorption was at hour four and it can provide valuable information for distinguishing malabsorption from non-compliance using a cut of less than 60% [19]. There are many reports of absolute LT4 absorption as low as 63% and as high as 195% by a similar equation [23-26]. LT4 absorption appears to be more highly absorbed in the hypothyroid patient compared to the normal individual [27]. In the present study, we tried to use the simplest possible protocol with the measurements of TT4 after two hours. Despite this factor, seven out of 17 patients with ≥60 % absorption had malabsorption on the long-supervised LT4 absorption test.

In the present study, we found that taking the 2-HR increment in FT4 and to a lesser extent TT4 had significant diagnostic accuracy to differentiate LT4 malabsorption and non-compliance. And a better result was seen when both FT4 and TT4 increments were taken together. Another study by Walker et al., 2013, included 23 patients with TSH refractory hypothyroidism showing a mean increment in FT4 after two hours about 54% ± 3% from baseline FT4 occurring in all patients in contrast to only 75% of patients showing improvement of TSH and not normalization after the fourth week of treatment with weight-related weekly doses of LT4. But these weekly doses were significantly lower than the equivalent daily dose which was already taken by patients before the study. In this study, there is no cut-off value to warrant investigating malabsorption. They conclude that using combined tests (long and rapid) significantly differentiates between malabsorption and non-compliance [17].

 Moreover, another study by Ghosh et al. depends on the absolute increment in FT4 during three hours of rapid LT4 absorption test and considers an increment ≥0.4 ng/dl (5.14 pmol/l) for non-compliance with a sensitivity of 97% and specificity of 80% to exclude malabsorption [22]. In this study, they did not use three hours of FT4/baseline FT4 ratio to assess the efficiency of LT4 absorption for two reasons. First, high baseline FT4 as the patient took a dose of LT4 the day of the test made a low ratio of three hours FT4/baseline FT4 and missed non-compliance. Second, in chronic prolonged hypothyroidism whose baseline FT4 can be very low, the three hours FT4/baseline FT4 ratio will be high and falsely diagnose non-compliance. However, in the present study in all of the 22 patients, the baseline hormonal measurements were done without morning intake of LT4 and still, the 2-HR FT4/baseline FT4 ratio failed to differentiate between non-compliance and malabsorption.

The study has limitations. First, there is no well-established gold standard for the differentiation between non-compliance and malabsorption. However, in the present study, we relied on the supervised long LT4 absorption test as a comparator. Second, the study was limited to a small sample and some of the included patients did not complete the overall five weeks follow-up.

## Conclusions

In conclusion, the rapid LT4 absorption test is a simple and safe test in the evaluation of patients with TSH refractory hypothyroidism. It showed a good diagnostic accuracy in the differentiation between non-compliance and malabsorption when 2-HR FT4 minus baseline FT4 and 2-HR TT4 minus baseline TT4 were used as criteria. The 2-HR FT4/baseline FT4 ratio and percentage absorption both lack diagnostic accuracy.
